# Vector Phase Analysis Approach for Sleep Stage Classification: A Functional Near-Infrared Spectroscopy-Based Passive Brain–Computer Interface

**DOI:** 10.3389/fnhum.2021.658444

**Published:** 2021-04-30

**Authors:** Saad Arif, Muhammad Jawad Khan, Noman Naseer, Keum-Shik Hong, Hasan Sajid, Yasar Ayaz

**Affiliations:** ^1^School of Mechanical and Manufacturing Engineering, National University of Sciences and Technology, Islamabad, Pakistan; ^2^National Center of Artificial Intelligence (NCAI), Islamabad, Pakistan; ^3^Department of Mechatronics Engineering, Air University, Islamabad, Pakistan; ^4^School of Mechanical Engineering, Pusan National University, Busan, South Korea

**Keywords:** functional near-infrared spectroscopy, brain-computer interface, drowsiness detection, vector phase analysis, cerebral oxygen regulation, sleep stages, multiclass classification, feature selection

## Abstract

A passive brain–computer interface (BCI) based upon functional near-infrared spectroscopy (fNIRS) brain signals is used for earlier detection of human drowsiness during driving tasks. This BCI modality acquired hemodynamic signals of 13 healthy subjects from the right dorsolateral prefrontal cortex (DPFC) of the brain. Drowsiness activity is recorded using a continuous-wave fNIRS system and eight channels over the right DPFC. During the experiment, sleep-deprived subjects drove a vehicle in a driving simulator while their cerebral oxygen regulation (CORE) state was continuously measured. Vector phase analysis (VPA) was used as a classifier to detect drowsiness state along with sleep stage-based threshold criteria. Extensive training and testing with various feature sets and classifiers are done to justify the adaptation of threshold criteria for any subject without requiring recalibration. Three statistical features (mean oxyhemoglobin, signal peak, and the sum of peaks) along with six VPA features (trajectory slopes of VPA indices) were used. The average accuracies for the five classifiers are 90.9% for discriminant analysis, 92.5% for support vector machines, 92.3% for nearest neighbors, 92.4% for both decision trees, and ensembles over all subjects’ data. Trajectory slopes of CORE vector magnitude and angle: *m*(|*R*|) and *m*(∠*R*) are the best-performing features, along with ensemble classifier with the highest accuracy of 95.3% and minimum computation time of 40 ms. The statistical significance of the results is validated with a *p*-value of less than 0.05. The proposed passive BCI scheme demonstrates a promising technique for online drowsiness detection using VPA along with sleep stage classification.

## Introduction

Invasive and noninvasive techniques are used in brain–computer interface (BCI) for the detection and measurement of brain activities using different BCI modalities (Sun et al., 2020; Tortora et al., 2020). Invasive BCI is based upon placing electrodes inside the brain cortex under direct interaction with neurons and hence requires complex surgery, medical conditions, and greater risk of infections (Yoo et al., 2018; Alkawadri, 2019; Romanelli et al., 2019). Nowadays, partially invasive techniques like electrocorticography (ECoG) are more in use. In ECoG, the electrode array is placed inside the skull and directly above the cortex. It requires easier surgery, and medical conditions like the infectious risk are very less (Romanelli et al., 2019). Furthermore, it provides the best signal quality, and good temporal and spatial resolution (Volkova et al., 2019). However, the availability of subjects is still a difficult task for invasive BCI techniques. Contrarily, noninvasive BCIs are more commonly used due to no surgery requirements and the absence of medical risks (Sosnik and Ben Zur, 2020).

Noninvasive BCIs use either electrophysiological signal or hemodynamic response phenomenon-based modalities. Electrophysiological BCI modalities are electroencephalography (EEG), electrooculography (EOG), electrocardiography (ECG), and electromyography (EMG), which record neuronal brain activity, eye movement, heart rate, and muscle movement, respectively (Turnip et al., 2011; Nicolas-Alonso and Gomez-Gil, 2012; Nguyen et al., 2017). Hemodynamic response-based modalities use functional neuroimaging models like functional near-infrared spectroscopy (fNIRS) and functional magnetic resonance imaging (fMRI), which record brain activity from changes in blood flow and blood oxygen levels in the active areas due to neuronal firing (Ni et al., 2017; Lasek-Bal et al., 2018; Yoo et al., 2018; Al-Zubaidi et al., 2019). Another functional BCI modality is magnetoencephalography (MEG), which is based upon recording the magnetic field in response to the electrical activity of neurons at active regions of the brain (Welvaert and Rosseel, 2014; Naseer and Hong, 2015). EEG and fNIRS BCIs are more widely used in detecting brain activity due to their low cost and better performance features (Borragan et al., 2018; Wascher et al., 2018; Rupp et al., 2019). Hybrid BCIs are also used, which include combinations of EEG, fNIRS, ECG, EOG, EMG, or other techniques depending upon which activities are to be recorded simultaneously for a specific task (Ahn et al., 2016; Choi et al., 2017; Nguyen et al., 2017; Hong et al., 2018). Among functional techniques, fNIRS is more safe, reliable, low-cost, portable, and easy to set up and has a good spatial resolution (Wolpaw et al., 2002; Ramadan and Vasilakos, 2017; Zhao et al., 2018). It measures changes in concentration of oxygenated hemoglobin (Δ*HbO*), deoxygenated hemoglobin (Δ*HbR*), total hemoglobin or cerebral blood volume (Δ*HbT*), and cerebral oxygen exchange (Δ*COE*) as a measure of brain activity in active regions resulted from neuronal consumption of glucose, measured by optical sensors using near-infrared light signals that are directly introduced into the scalp, and hence, it is free from noise and electrical interference (Tanveer et al., 2019; Wang et al., 2019; Khan M.N.A. et al., 2020; Khan R.A. et al., 2020).

Brain activities are recorded and classified under active, reactive, and passive states of BCI (Zander and Kothe, 2011). Active BCI records brain activity generated due to intentional actions like mental computation tasks, motor imagery, and motion intents. Reactive BCI records brain activity produced in response to some external stimuli like audio, video, touch, or pain signal introduction (Hong and Khan, 2017; Khan and Hong, 2017; Khan M.J. et al., 2018). Active and reactive brain signals can be more easily generated and detected, unlike passive brain activities. Passive brain activities are produced unintentionally by a human brain under certain body conditions like drowsiness, sleep, fatigue, stress, loss of attention, or focus (Khan and Hong, 2015; Tanveer et al., 2019; Dunbar et al., 2020). These passive states imply very crucial effects when arising during high attention-seeking tasks like vehicle driving. Drowsiness or sleep during driving causes severe accidents worldwide (Philip and Åkerstedt, 2006; Ismail et al., 2009). Conventional techniques to detect drowsiness may include measuring the eye blink rate, heart rate, or head movement with increased chances of false detections. However, a passive BCI system is more preferred to detect drowsiness conditions from brain signals, and an activity can be well estimated earlier and in a precise manner. fNIRS-based BCI is also used to detect brain states due to fatigue or sleep loss (Ioannides, 2018). This activity is recorded from the prefrontal cortex (PFC) and specifically from dorsolateral PFC (DPFC) (Khan and Hong, 2015; Tanveer et al., 2019). Studies have shown rapid and increased brain activity in the DPFC region under brain state transitions from wakefulness to non-rapid eye movement (NREM) sleep stages (Khan and Hong, 2015; Bernardi et al., 2018). This results in increased concentrations of Δ*HbO* and decreases in Δ*COE*, which indicates sleep as a refreshing process (Khero et al., 2019; Oniz et al., 2019). During driving, these rapid changeovers between sleep stages (as experienced when a person is consistently nodding off) could be devastating and must be recorded at an earlier stage to avoid life losses (Fonseca et al., 2018; Dai et al., 2020).

This study investigates a novel drowsiness detection scheme using hemodynamic activities of the brain with a passive BCI. Hemodynamic brain signals are acquired from the right DPFC using eight channels of the fNIRS system. All the hemodynamic signals are plotted upon vector phase analysis (VPA) to get the cerebral oxygen regulation (CORE) status of the brain. Sleep stage-based threshold circles are employed on VPA, which resulted from systematically proposed criteria. The criteria deduce radii of sleep stage (N1, N2, and N3) threshold circles from sample data of wakefulness (W) stage of the subject. As CORE status is constantly monitored over VPA against threshold circles, drowsiness activity is detected when the CORE trajectory crosses threshold circles in specific octants of VPA. The universality and validity of proposed threshold circle criteria for any subject is the core and fundamental objective of this work. The criteria of threshold circles are validated over fNIRS data of 13 subjects. A total of nine features are used for training and classification, out of which six features are extracted from VPA and three statistical features from Δ*HbO* signal. Five machine learning classifiers [discriminant analysis (DA), support vector machines (SVM), decision trees (DT), k-nearest neighbors (kNN), and ensembles] are used to classify the data of all the subjects according to the proposed scheme. Slopes of CORE vector magnitude and angle are the best feature pair along with the SVM classifier to perform well overall.

## Materials and Methods

### Subjects/Participants

To collect this drowsiness dataset (Khan and Hong, 2015; Tanveer et al., 2019), 13 healthy male subjects (mean age: 28.5 ± 4.8 years) were recruited. Two of them were left-handed, and all had normal or corrected-to-normal vision. Neither of them was reported to have any psychiatric, visual, or neurological disorder. All the participants willingly consented when details about the experimental procedure were explained. The study was reviewed and approved by Pusan National University Institutional Review Board.

### Experimental Procedure

**FIGURE 1:**
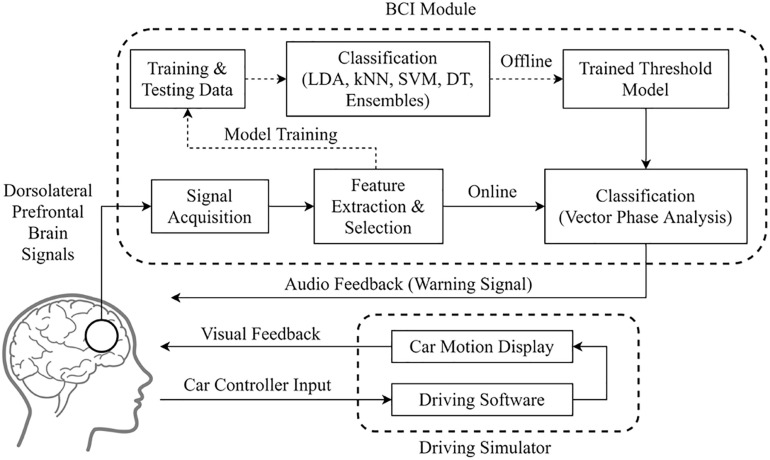
Experimental setup and its flowchart for drowsiness detection.

The experiment was conducted in the morning before which all subjects were sleep-deprived for 10 h the night before. Participants were subjected to car driving in a simulated environment with medium traffic and pedestrian density. Brain signals for 5 min were collected for baseline adjustment during initial driving trials for environment familiarization. Each subject drove the car for almost 1 h in which fNIRS signals were collected for 30 ± 5 min when they were visually observed to be near drowsy. Biomarkers for the drowsy state were placed when a change in facial expressions or eye closure was observed due to sleep loss or fatigue. Subjects remained seated in comfortable chairs and were asked to minimize head or muscle movements to avoid motion-related artifacts in brain signals. Figure 1 shows the flow diagram of the experimental procedure.

### Sensor Configuration

**FIGURE 2:**
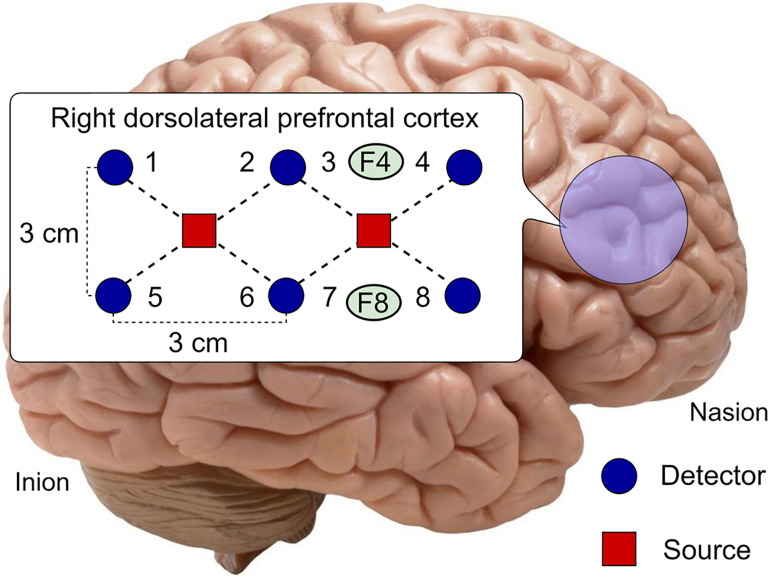
Optode placement at Region A (right dorsolateral prefrontal cortex), channels 1–8.

Seven sources with 16 detectors of the near-infrared range were used to make combinational pairs of 28 channels to acquire fNIRS signals over various brain locations. Optodes for these 28 channels were placed at PFC and DPFC according to the international 10–20 system. The distance between adjacent detectors was 3 cm, and the distance between source and detector was 2.1 cm. These 28 channels were further divided into three regions (A, B, and C). Region A comprises channels 1–8, which were placed at the right DPFC as shown in Figure 2. Channels 9–20 were regarded as region B and placed at PFC. Channels 21–28 were placed at left DPFC and specified as region C. Right DPFC (region A) is proved to be more suitable and effective for drowsiness-related activity detection (Khan and Hong, 2015; Tanveer et al., 2019). In this research work, fNIRS data from channels 1–8 (region A) are focused on sleep detection in online passive BCI applications.

### Signal Acquisition and Processing

A continuous-wave imaging system (DYNOT, NIRx Medical Technologies, United States) was used for fNIRS brain signal acquisition. Data were obtained at a sampling frequency of 1.81 Hz with near-infrared lights of 760 and 830 nm wavelengths. Motion-related and other artifacts were removed from the acquired data by applying Gaussian filters (Bhutta et al., 2014; Khan and Hong, 2015; Zafar and Hong, 2018). Band rejection of ranges 0.3∼0.4, 1∼1.2, and <0.01 Hz were used for respiration, heartbeat, and Mayer-wave artifact removal, respectively. Oxygenated and deoxygenated hemoglobin concentration changes (Δ*HbO* and Δ*HbR*, respectively) were obtained by converting raw intensity values of two different wavelengths by using modified Beer–Lambert law (MBLL). The MBLL is stated as,

(1) $$A\,\left(t;\lambda \right)=\ln\left(\frac{I_{i\,n}\,\left(\lambda \right)}{I_{o\,u\,t}\,\left(t;\lambda \right)}\right)=\alpha \,\left(\lambda \right)\,\times \,c\,\left(\lambda \right)\,\times \,l\times \,d\,\left(\lambda \right)+\eta$$

(2) $$\left[\begin{array}{c} \Delta \,c_{H\,b\,O}\,\left(t\right)\\ \Delta \,c_{H\,b\,R}\,\left(t\right) \end{array}\right]={\left[\begin{array}{cc} \alpha_{H\,b\,O}\,\left(\lambda_{1}\right)\,\alpha_{H\,b\,R}\,\left(\lambda_{1}\right) & \\ \alpha_{H\,b\,O}\,\left(\lambda_{2}\right)\,\alpha_{H\,b\,R}\,\left(\lambda_{2}\right) & \end{array}\right]}^{-1}\,\left[\begin{array}{c} \Delta \,A\,\left(t;\lambda_{1}\right)\\ \Delta \,A\,\left(t;\lambda_{2}\right) \end{array}\right]\,\frac{1}{l\times \,d\,\left(\lambda \right)}$$

where *A* is the absorbance of light (optical density), *I*_*in*_ is the incident intensity of light, *I*_*out*_ is the detected density of light, α is the specific extinction coefficient in μM^–1^ cm^–1^, *c* is the absorber concentration in μM, *l* is the distance between the source and the detector in cm, *d* is the differential path-length factor (DPF), and η is the loss of light due to scattering.

### Vector Phase Analysis

**FIGURE 3:**
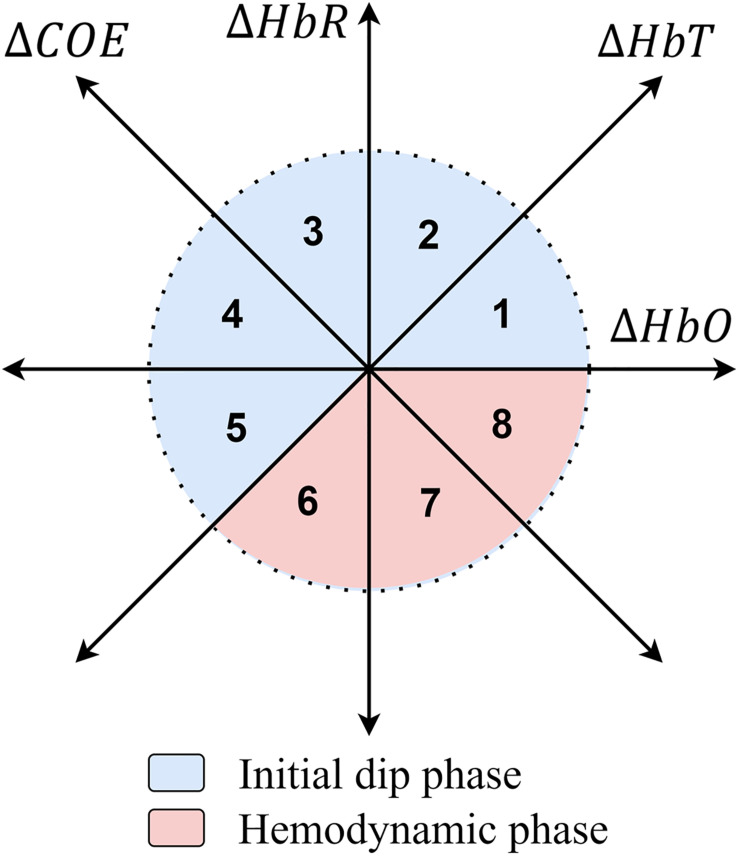
Vector phase diagram (Hong and Naseer, 2016).

If the hemodynamic indicators of fNIRS signals (Δ*HbO* and Δ*HbR*) are mapped as orthogonal axes in an orthogonal vector coordinate plane, then they give rise to a very promising scheme regarded as VPA method as shown in Figure 3. When this orthogonal coordinate plane is rotated by an angle of π /4 rad counterclockwise, then it adds up new useful components in this vector plane: Δ*HbT* and Δ*COE* (due to neurovascular coupling) (Yoshino and Kato, 2012; Hong and Naseer, 2016; Hong et al., 2018; Zafar and Hong, 2018; Nazeer et al., 2020a). These indices are defined as,

(3) Δ⁢H⁢b⁢T=Δ⁢H⁢b⁢O+Δ⁢H⁢b⁢R

(4) Δ⁢C⁢O⁢E=Δ⁢H⁢b⁢R-Δ⁢H⁢b⁢O

The relationship among all these four hemodynamic indices is given in the following mathematical notation.

(5) $$\left[\begin{array}{c} \Delta \,H\,b\,O+\Delta \,H\,b\,R\\ -\Delta \,H\,b\,O+\Delta \,H\,b\,R \end{array}\right]=\left[\begin{array}{cc} 11 & \\ -11 & \end{array}\right]\,\left[\begin{array}{c} \Delta \,H\,b\,O\\ \Delta \,H\,b\,R \end{array}\right]=\left[\begin{array}{c} \Delta \,H\,b\,T\\ \Delta \,C\,O\,E \end{array}\right]$$

(6) $$\left[\begin{array}{c} \Delta \,H\,b\,O\\ \Delta \,H\,b\,R \end{array}\right]=\frac{1}{2}\,\left[\begin{array}{cc} 1-1 & \\ 11 & \end{array}\right]\,\left[\begin{array}{c} \Delta \,H\,b\,T\\ \Delta \,C\,O\,E \end{array}\right]$$

Any point on this vector coordinate plane holds a value-based upon four indices Δ*HbO*, Δ*HbR*, Δ*HbT*, and Δ*COE*; and its distance from the origin specifies a vector *R* that reveals information about CORE (Yoshino and Kato, 2012; Hong and Naseer, 2016; Nazeer et al., 2020a). The magnitude |*R*| and angle ∠*R* of vector *R* are stated below.

$$\left|R\right|=\sqrt{{\left(\Delta \,H\,b\,O\right)}^{2}+{\left(\Delta \,H\,b\,R\right)}^{2}}$$

$$=\frac{1}{\sqrt{2}}\,\sqrt{{\left(-\Delta \,H\,b\,O+\Delta \,H\,b\,R\right)}^{2}+{\left(\Delta \,H\,b\,O+\Delta \,H\,b\,R\right)}^{2}}$$

(7) $$=\frac{1}{\sqrt{2}}\,\sqrt{{\left(\Delta \,C\,O\,E\right)}^{2}+{\left(\Delta \,H\,b\,T\right)}^{2}}$$

(8) $$∠\,R=\tan^{-1}\left(\frac{\Delta \,H\,b\,R}{\Delta \,H\,b\,O}\right)=\tan^{-1}\left(\frac{\Delta \,C\,O\,E}{\Delta \,H\,b\,T}\right)+\frac{\pi }{4}$$

Based upon axes of this vector coordinate plane, it is divided into eight octants, each of which represents specific hemodynamic features of the fNIRS brain signal as shown in Figure 3. These octants are referred to as phases indicating oxygenated (oxic) and deoxygenated (capnic) states of the brain. Increased and decreased blood oxygenation refers to hyperoxic (HerOx) and hypoxic (HyOx) states (Dart et al., 2017), while increased and decreased blood deoxygenation refers to hypercapnic (HerCap) and hypocapnic (HyCap) states of the brain, respectively (Smith et al., 2017). All these CORE states and hemodynamic signal features in these phases are tabulated in Table 1.

**TABLE 1:** Characteristics of different phases in the vector phase diagram.

Phases	Δ*HbO*	Δ*HbR*	Δ*HbT*	Δ*COE*	Condition	CORE state	Signal feature
1	Positive	Positive	Positive	Negative	Δ*HbO* > Δ*HbR*	*HerOx*≫*HerCap*	Initial dip
2	Positive	Positive	Positive	Positive	Δ*HbO* < Δ*HbR*, Δ*HbT* > Δ*COE*	*HerOx*≪*HerCap*	
3	Negative	Positive	Positive	Positive	Δ*HbT* < Δ*COE*	*HyOx*≪*HerCap*	
4	Negative	Positive	Negative	Positive		*HyOx*≫*HerCap*	
5	Negative	Negative	Negative	Positive	Δ*HbO* < Δ*HbR*	*HyOx*≫*HyCap*	
6	Negative	Negative	Negative	Negative	Δ*HbO* > Δ*HbR*,Δ*HbT* < Δ*COE*	*HyOx*≪*HyCap*	Hemodynamic activity
7	Positive	Negative	Negative	Negative	Δ*HbT* > Δ*COE*	*HerOx*≪*HyCap*	
8	Positive	Negative	Positive	Negative	Δ*HbT* < Δ*COE*	*HerOx*≫*HyCap*	

*CORE, cerebral oxygen regulation.*

### Sleep Stage-Based Threshold Circles

Long sleep deprivation may cause brain sleep or hallucinations while a person seems awake. In such cases, drowsiness can instantly lead the human brain through various sleep stages (Craik et al., 2019; Ko et al., 2020). Moreover, in such a condition of drowsiness, if the brain is being forced to focus, then it can result in frequent state changeovers between wakefulness and sleep stages, for example, repetitively nodding off while driving in a drowsy state. Drowsiness is interrelated with sleep in terms of physical symptoms and effects on human brain hemodynamics. Sleep stages are NREM sleep and rapid eye movement (REM) sleep. NREM has further three stages: N1, N2, and N3 representing light sleep, medium sleep, and deep sleep, respectively (Ahn et al., 2016; Oniz et al., 2019). The sleep cycle starts when wakefulness is followed by NREM stages and REM sleep (Van Wyk et al., 2019; Chi et al., 2020). The main feature of drowsiness is slow rolling eye movements (SREM), which are associated with the N1 stage or light sleep. fNIRS studies have investigated brain hemodynamics during various sleep stages and discussed CORE dynamics related to sleep (Oniz et al., 2019). It is conceived that there is a relationship between hemodynamics of sleep stages and W. If CORE dynamics of W is known, then CORE of sleep stages can be deduced from it according to a somewhat fixed relationship as shown in (10). Mean CORE status during W of any subject can be accessed by collecting |*R*| of fNIRS brain signal for a specific duration and taking its mean. Mean |*R*| of NREM sleep stages can be deduced from mean |*R*| of W stage according to the following relationships.

(9) $$\bar{\left|R_{K}\right|}=\frac{1}{n}\,\sum_{i=\,1}^{n}\sqrt{{\left(\Delta \,H\,b\,O_{K}\right)}_{i}^{2}+{\left(\Delta \,H\,b\,R_{K}\right)}_{i}^{2}},\,s.t.K=\,\left[W\,\,N\,1\,\,N\,2\,\,N\,3\right]$$

(10) $$\left[\begin{array}{c} \bar{\left|R_{N\,1}\right|}\\ \bar{\left|R_{N\,2}\right|}\\ \bar{\left|R_{N\,3}\right|} \end{array}\right]=\left[\begin{array}{c} 0.8778\\ 0.8077\\ 0.6544 \end{array}\right]\,\times \,\bar{\left|R_{W}\right|}$$

where $\bar{\left|R_{K}\right|}$ represents $\bar{\left|R_{W}\right|}$,$\bar{\left|R_{N\,1}\right|}$,$\bar{\left|R_{N\,2}\right|}$,$\bar{\left|R_{N\,3}\right|}$, which are the sample means of phase diagram vectors’ magnitude for W, N1, N2, and N3, stages, respectively; and *n* is the number of samples used for mean values computation from the respective sample spaces.

**FIGURE 4:**
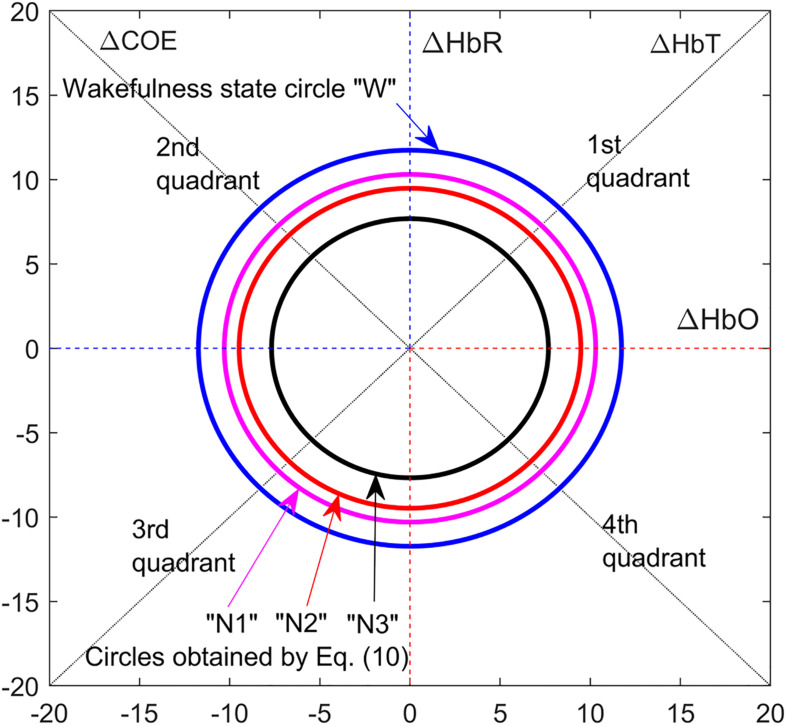
Threshold circles of wakefulness and non-rapid eye movement (NREM) sleep stages employed for drowsiness detection and sleep stage classification, obtained from Eqs. (9, 10).

Eqs. (9, 10) give the radii of threshold circles for W and NREM stages in the vector phase diagram as shown in Figure 4. These threshold circles along with the VPA diagram are employed to detect drowsiness activity when the fNIRS brain signal trajectory follows a specific pattern according to CORE states. Eq. (9) is evaluated for all eight channels of right DPFC for each subject with sample space spanning over 5 min of W state. Once $\bar{\left|R_{W}\right|}$ is obtained, Eq. (10) is evaluated for all channels of each subject to obtain $\bar{\left|R_{N\,1}\right|}$,$\bar{\left|R_{N\,2}\right|}$,$\bar{\left|R_{N\,3}\right|}$.

### Vector Phase Analysis Trajectory Pattern for Drowsiness Detection

During wakefulness, focus/attention-seeking tasks, neurons consume more glucose, resulting in increased Δ*COE*, and the brain experiences HerCap as well as HyOx CORE states (Skalski and Dobrakowski, 2020; Tung et al., 2020). So the VPA trajectory mostly remains in phases 3 and 4 of the vector phase diagram (second quadrant). Loss of focus/attention or drowsiness results in a decrease in mental activity, and hence, a decrease in Δ*COE* is observed. The brain experiences HerOx as well as HyCap CORE states, and the VPA trajectory mostly remains in phases 7 and 8 of the vector phase diagram (fourth quadrant). It has been noted that transitioning from wakefulness to sleep increases the oxygen levels in the blood (Khero et al., 2019; Oniz et al., 2019), so Δ*HbO* rises as well as Δ*COE* falls gradually. Eventually, the brain has higher Δ*HbO* and lower Δ*HbR* levels by the end of sleep (Oniz et al., 2019). The key to early detection of drowsiness activity is to capture this CORE state transition from HyOx and HerCap to HerOx and HyCap states (Chuang et al., 2018; Lin et al., 2020). At each instance, CORE state points are plotted upon vector phase diagram using Eqs. (7, 8), and a trajectory is obtained. To detect drowsiness activity, an angle criterion and a magnitude criterion for this trajectory are defined as shown below.

(11) $$\frac{3}{4}\pi <\left(\bar{∠\,R}=\frac{1}{n}\sum_{i=\,1}^{n}{\left(∠R\right)}_{i}\right)<2\pi$$

(12) $$\left(\bar{\left|R\right|}=\frac{1}{n}\sum_{i=\,1}^{n}{\left(|R|\right)}_{i}\right)>\bar{\left|R_{W}\right|}$$

where *n* is the number of samples required to find the mean angle and mean magnitude for the duration of 0–5 s time window, which is reported to be the shortest to find the drowsiness activity (Khan and Hong, 2015). It is to be noted that the angle criterion is of primary importance here, and it must be satisfied before the magnitude criterion holds. Drowsiness activity is successfully detected when relationships (11) and (12) hold and the VPA trajectory crosses the threshold circle of W state in phases 7 and 8 of the vector phase diagram as shown in the section “Results.”

### Feature Space and Classification

To standardize the threshold circle criteria as a standard framework for any subject to detect activity online, comprehensive testing and validation are need (Koo and Choi, 2020). So to further test and justify this criterion based on sleep stages, multiclass classification is done with various classifiers and different feature spaces (Kang et al., 2020; Sung et al., 2020). As drowsiness detection is intended from VPA, six features were selected by keeping in view the promising information of vector phase diagram, which are trajectory slopes of VPA indices and parameters; *m*(Δ*HbO*), *m*(Δ*HbR*), *m*(Δ*HbT*), *m*(Δ*COE*), *m*(∠*R*), and *m*(|*R*|). Other three statistical features were extracted from Δ*HbO* time signal, namely, signal mean (*M*), signal peak (*P*), and the sum of peaks (*SoP*), which were reported to be the best suitable features of fNIRS signal for binary class classification of drowsiness activity (Khan and Hong, 2015; Tanveer et al., 2019; Nazeer et al., 2020a). All features were computed for the 0–5 s time window for all subjects according to the following relations.

(13) $$m\,\left(\Delta \,X_{k}\right)=\frac{X_{N}-X_{1}}{\text{length}\,\left(k\right)},\,s.t.\,k=\,1,\ldots ,N$$

(14) $$M_{k}=\frac{1}{N}\,\sum_{i=\,1}^{N}\Delta \,H\,b\,O_{i}$$

where *k* is the sample vector for 0–5 s time window with *N* as the last sample in it, *X* is the variable for six parameters of the vector phase diagram and *m*(Δ*X*) is slope or gradient of these parameters over *k*, and *M*_*k*_ is the mean value of Δ*HbO* in *k*. *P* was calculated as the maximum value of local maximums of Δ*HbO* in *k* by using *max* and *findpeaks* functions of MATLAB 9.5 (MathWorks, United States). *SoP* was computed as a summation of local maximums calculated above.

After feature extraction, feature scaling was done in the range [a  b]=[−1    1] for all features by using min–max normalization as stated below.

(15) $$Y^{{}^{\prime }}=a+\frac{Y-\text{min}\,\left(Y\right)}{\text{max}\,\left(Y\right)-\text{min}\,\left(Y\right)}\,\left(b-a\right)$$

where *Y* is original value and *Y*′ is rescaled/normalized value of the feature in the said range.

These rescaled features are further employed in multiclass classifiers for training and testing the dataset using DT, DA, SVM, kNN (Zhu et al., 2019; Ali and Park, 2020; Kim et al., 2020; Li et al., 2020), and ensemble classifiers. For better classification of samples in the respective classes, the value of k is set to 1 in the kNN classifier. To cater to the nonlinearity with SVM classifier, fine Gaussian SVM is used with radial basis function kernel and c=0.5. The dataset comprises all observations from all channels of region A for all subjects. For training and testing of the data and to analyze the performance of all classifiers, 10-fold cross-validation is used to find an optimal separation between W, N1, N2, and N3 stages in this four-class classification problem. The multiclass classification performance evaluation is validated with the help of the confusion matrix, receiver operating characteristic (ROC) curves, and their area under the curve (AUC). The parameters true-positive rate (TPR), false-negative rate (FNR), true-negative rate (TNR), false-positive rate (FPR), positive predictive value (PPV), and false discovery rate (FDR) are calculated to find out sensitivity, miss rate, specificity, fall-out, and precision using following equations, respectively.

(16) $$T\,P\,R=\frac{T\,P}{T\,P+F\,N}=1-F\,N\,R$$

(17) $$F\,N\,R=\frac{F\,N}{F\,N+T\,P}=1-T\,P\,R$$

(18) $$T\,N\,R=\frac{T\,N}{T\,N+F\,P}=1-F\,P\,R$$

(19) $$F\,P\,R=\,\frac{F\,P}{F\,P+T\,N}=1-T\,N\,R$$

(20) $$P\,P\,V=\frac{T\,P}{T\,P+F\,P}=1-F\,D\,R$$

(21) $$F\,D\,R=\frac{F\,P}{F\,P+T\,P}=1-P\,P\,V$$

## Results

**FIGURE 5:**
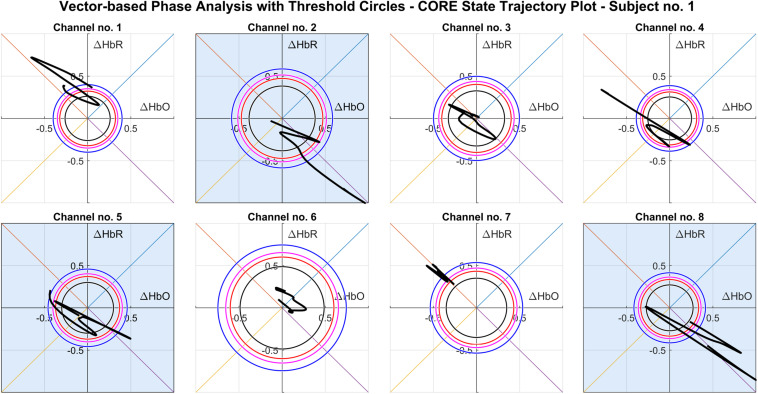
Vector phase analysis (VPA) trajectories of all channels (Subject 1) obtained by plotting Eqs. (7, 8) at the drowsiness stage. The shaded boxes show the active detection channels in which trajectory has crossed the W threshold circle in the fourth quadrant according to the magnitude and angle criterion.

This study proposes a novel online classification technique for the early detection of driver drowsiness using VPA. The outermost threshold circle is based on the mean CORE vector magnitude of the wakefulness state $\bar{\left|R_{W}\right|}$, which can vary subject-wise and can be deduced from initial baseline data. For this purpose, five trials per subject were performed at different time instants, where each trial period was 5 min followed by significant rest time to avoid fatigue effect. While inner threshold circles are based on mean CORE vector magnitude of NREM sleep stages N1, N2, and N3, $(\bar{\left|R_{N\,1}\right|}$,$\bar{\left|R_{N\,2}\right|}$,$\bar{\left|R_{N\,3}\right|})$ and are dependent upon the W circle according to a fixed relationship as in Eq. (10). CORE state points based upon Δ*HbO* and Δ*HbR* are continuously being plotted over vector phase diagram, and a continuous VPA trajectory is obtained as shown in Figure 5. Drowsiness activity is detected when the VPA trajectory crosses the W state threshold circle in phases 7 and 8 of the vector phase diagram according to the criterion of Eqs. (11, 12), showing a decrease in Δ*COE*, which is an indicator of transition from W to NREM sleep. Trajectory computation and its slope assessment are done over a margin of 5 s to avoid false detection through this scheme. The results obtained using the proposed scheme for all the channels of region A of Subject 1 are shown in Figure 5. The active channels are highlighted with shaded boxes in which a trajectory crosses the threshold circles in the fourth quadrant of the phase diagram, indicating drowsiness activity detection.

**FIGURE 6:**
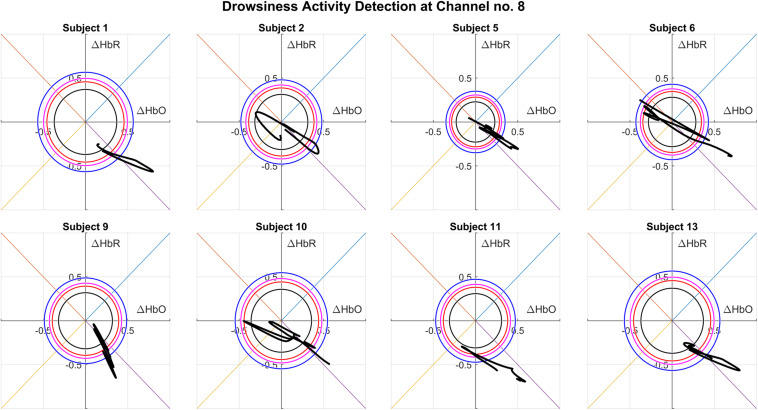
Vector phase analysis (VPA) trajectories of various subjects at an active channel (Channel 8) showing drowsiness activity detection, located at F8 electrode position of the 10–20 system.

The active channels and consequently the precise brain region for drowsiness detection can be identified using this proposed novel scheme. Upon further assessment of active channels for all the subjects, Channel 8 turns out to be the most active channel among all and is situated near the F8 electrode position according to the 10–20 system. Figure 6 shows the successful drowsiness detection on Channel 8 of various subjects. It is to be noted that trajectory patterns are not the same as the active channel of different subjects. Trajectories could follow any path, but they must satisfy the proposed angle and magnitude criterion. Real-time signals of Δ*HbO* and Δ*HbR* could be used according to the proposed scheme to detect drowsiness online as soon as the onset of activity.

The computation of sleep stage thresholds for any subject requires extensive system training and time-taking assessment each time. To avoid this need for retraining and reassessment, a universally applicable criterion is easier to follow each time for any subject with minimum system training requirements. In this study, such a criterion is proposed, which is universally applicable to any subject by only requiring baseline or reference data of wakefulness or resting state. The applicability of sleep stage-based threshold circle criteria for any subject needed extensive validation, which makes it a standard scheme in online detection systems.

**FIGURE 7:**
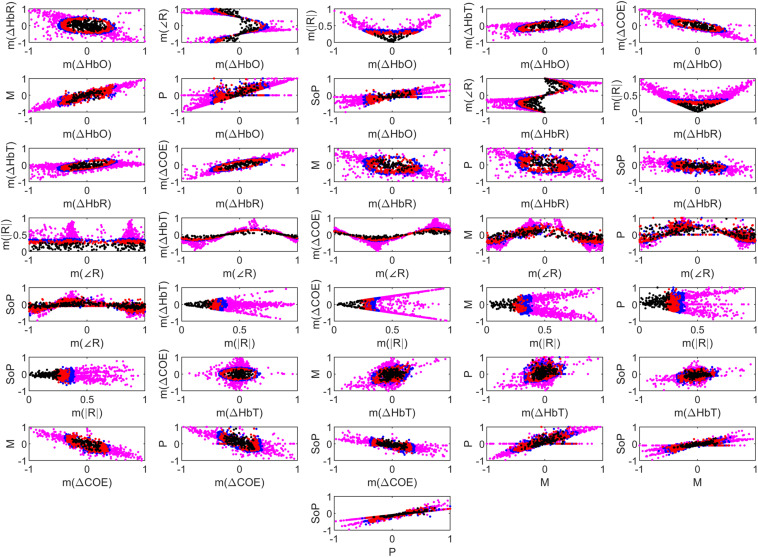
Thirty-six 4-class feature spaces combining all features [six vector phase analysis (VPA) and three statistical] for separating the W, N1, N2, and N3 stages represented by pink, blue, red, and black colored data points, respectively.

For this purpose, multiple classifiers were trained and tested for all possible combinations of two features. Figure 7 presents 36 two-dimensional feature spaces for all feature combinations calculated for the 0–5 s time window over all 13 subjects. It can be observed that *m*(Δ*HbR*) vs. *m*(Δ*HbO*), *m*(|*R*|) vs. *m*(∠*R*) and [*m*(Δ*HbT*),*m*(Δ*COE*),*P*] vs. *m*(|*R*|) provide the best data separation between sleep stages. Table 2 shows the classification accuracies obtained with all possible binary pairs of features using best-performing classifiers among the five mentioned above. So the above-mentioned feature combinations resulted in 90% classification accuracy, while all other pairs resulted in an accuracy of 70% and above, except only three pairs between 65 and 70%. Table 2 further supports confidence in using features based on all six VPA indices for sleep stage classification.

**TABLE 2:** Percentage accuracies obtained by combinations of two features (0–5 s window, all subjects, kNN classifier).

Features	*m*(Δ*HbR*)	*m*(∠*R*)	*m*(|*R*|)	*m*(Δ*HbT*)	*m*(Δ*COE*)	*M*	*P*	*SoP*
*m*(Δ*HbO*)	90.0	88.4	89.9	89.9	89.9	75.1	74.9	74.9
*m*(Δ*HbR*)	–	88.3	89.9	89.8	89.9	84.1	81.3	79.6
*m*(∠*R*)	–	–	90.0	84.7	88.0	74.7	70.3	68.3
*m*(|*R*|)	–	–	–	90.0	90.0	89.8	90.0	89.8
*m*(Δ*HbT*)	–	–	–	–	89.9	76.5	70.4	69.0
*m*(Δ*COE*)	–	–	–	–	–	85.8	83.7	82.1
*M*	–	–	–	–	–	–	71.8	71.4
*P*	–	–	–	–	–	–	–	65.5

Table 3 shows subject-wise classification accuracies obtained with the best-performing feature combination and classifier. It has been observed that the *m*(∠*R*), *m*(|*R*|) pair performed the best for 11 subjects out of 13, with an almost 85% success rate. For two subjects, *m*(Δ*HbO*),  *m*(Δ*HbR*) performed well comparatively but without significant improvement in the accuracy. So it can be deduced that trajectory gradients of CORE vector angle and magnitude are optimal VPA features for sleep stage classification, as they resulted in more than 90% accuracy for all subjects. Hence, drowsiness activity can be obtained by observing which phase the VPA trajectory lies in and what its distance is from the vector phase diagram’s origin. By constantly observing the relevant change in these two aspects, drowsiness activity can be detected.

**TABLE 3:** Best classification accuracies in brain region A (all channels, 0–5 s window, SVM classifier).

Subject	Accuracy (%)	Feature set
1	93.4	*m*(∠*R*),*m*(|*R*|)
2	91.4	*m*(Δ*HbO*),*m*(Δ*HbR*)
3	95.4	*m*(∠*R*),*m*(|*R*|)
4	93.2	*m*(∠*R*),*m*(|*R*|)
5	92.7	All six VPA features
6	93.4	*m*(Δ*HbO*),*m*(Δ*HbR*)
7	93.9	*m*(∠*R*),*m*(|*R*|)
8	90.5	*m*(∠*R*),*m*(|*R*|)
9	95.3	*m*(∠*R*),*m*(|*R*|)
10	91.0	All six VPA features
11	93.4	*m*(∠*R*),*m*(|*R*|)
12	91.5	*m*(∠*R*),*m*(|*R*|)
13	92.4	*m*(∠*R*),*m*(|*R*|)
Mean	92.9	*m*(∠*R*) and *m*(|*R*|) performed well overall

*SVM, support vector machine; VPA, vector phase analysis.*

Table 4 compares the percentage accuracy and computation time for cross-validated multiclass classifiers. It is noted that all the classifiers other than DA performed well with less variance accuracy among them but significant differences in computation time. SVM has the highest accuracy, but its computation time almost doubled as compared with that of DT and kNN classifiers. Ensemble classifier is chosen as the best-performing classifier because it has the least computation time and its accuracy is almost the same as that of SVM. Student’s *t*-test method is applied for the comparison of ensemble classifier’s accuracy with other classifier accuracies. Results show the *p*-value of less than 0.05 for all tests, which validated the statistical significance of the hypothesis made over outperforming ensemble classifier. Only the best-performing feature set “*m*(∠*R*) and *m*(|*R*|)” is used for classification accuracies obtained in Table 4.

**TABLE 4:** Performance comparison of five classifiers: accuracy (%)/computation time (s).

Subject	Decision trees	Discriminant analysis	Support vector machine	Nearest neighbor	Ensembles
1	94.4/0.102	92.0/0.151	92.8/0.208	93.0/0.138	93.4/0.057
2	91.0/0.105	89.6/0.154	90.5/0.207	91.6/0.135	91.1/0.045
3	94.7/0.091	94.5/0.140	94.6/0.200	94.4/0.131	95.3/0.040
4	93.6/0.100	89.0/0.141	91.9/0.203	91.3/0.130	93.0/0.042
5	91.8/0.105	89.5/0.145	92.2/0.211	92.5/0.134	92.2/0.041
6	92.3/0.102	92.7/0.146	93.1/0.212	92.6/0.130	92.9/0.039
7	93.5/0.103	93.8/0.141	93.4/0.218	94.3/0.140	93.7/0.044
8	89.2/0.102	88.7/0.142	91.0/0.212	88.9/0.131	89.4/0.049
9	92.6/0.109	91.0/0.140	94.0/0.223	95.4/0.133	94.1/0.046
10	89.0/0.106	90.8/0.146	90.5/0.213	89.4/0.138	88.8/0.042
11	93.6/0.107	91.7/0.146	93.1/0.203	93.6/0.124	93.1/0.041
12	92.4/0.097	87.3/0.135	92.2/0.204	91.0/0.129	92.1/0.041
13	92.4/0.100	91.3/0.140	92.8/0.203	92.0/0.132	92.2/0.041
Mean	92.4/0.102	90.9/0.144	92.5/0.209	92.3/0.133	92.4/0.044

**FIGURE 8:**
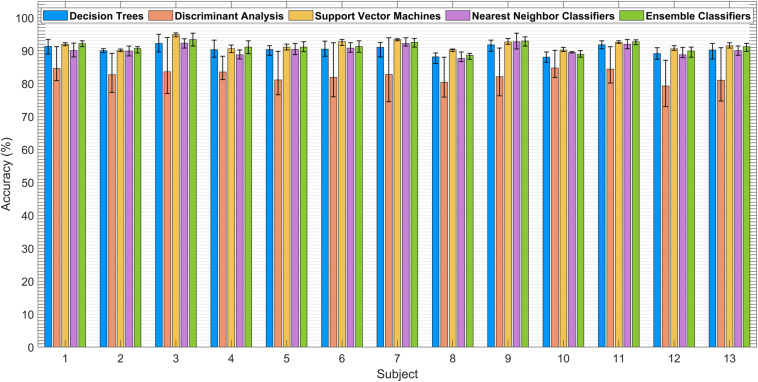
Average classification accuracies with variance bounds obtained by using different vector phase analysis (VPA) feature pairs.

Figure 8 presents average classification accuracies obtained with all five classifiers for all subjects. Variance in accuracy values due to the usage of multiple VPA feature combinations is showed by error bars against each classifier. Here, DA and SVM showed maximum and minimum sensitivity to change of features, respectively. The length of variance bounds shows the standard deviation (SD) of accuracy from the mean value. The SD is the highest in DA, which shows that accuracy changes significantly if the feature set changes, while SD for SVM is the lowest, which shows that accuracy is minimally affected when different feature sets are used for classification.

**FIGURE 9:**
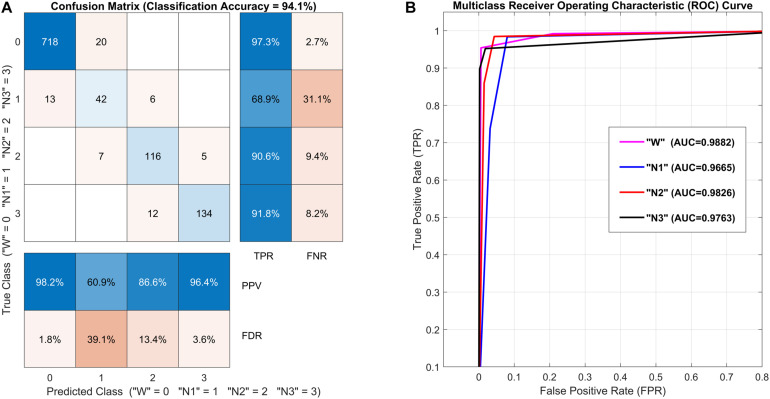
Classification performance measures [Subject 9, all channels, *m*(|*R*|) vs. *m*(∠*R*) feature set, ensemble classifier]: **(A)** Confusion matrix with the number of observations at diagonal and off-diagonal entries, true-positive rate (TPR), and false-negative rate (FNR) at the right columns, and positive predictive value (PPV) and false discovery rate (FDR) at the bottom rows. **(B)** Multiclass receiver operating characteristic (ROC) curves and area under the curve (AUC) for all sleep stages.

Figure 9 illustrates classification performance measures in terms of confusion/error matrix, ROC curves, and AUC. The ensemble classifier performed very well with an accuracy of 94.1% and AUC near 1 for all classes with a cross-validated classification model. Hence, these results increase the confidence in using the proposed criterion for any subject with minimum setup time.

## Discussion

In previous studies related to passive BCI systems, 0–5 and 0–1 s time windows were used to classify the loss of attention/vigilance from fNIRS brain signals with off-line classification techniques (Khan and Hong, 2015; Tanveer et al., 2019). However, less focus is given to real-time or online detection of passive brain states using fNIRS signals for real-life/practical applications. Studies have shown an increase in Δ*HbO* levels in the DPFC region of the brain when passive states are overcoming focus during attention-demanding tasks (Khan and Hong, 2015; Tanveer et al., 2019). An increase in Δ*HbO* and a decrease in Δ*COE* is also observed, as sleep is overcoming consciousness (Khero et al., 2019; Oniz et al., 2019). So the results of this study are consistent with the literature and support the hypothesis presented. Here, the trends of the brain’s CORE state are monitored from wakefulness to sleep, which gives a new scheme for the detection of drowsiness or sleep. The spatial resolution is also increased for the detection of passive activity from the right DPFC with Channel 8 (near F8 electrode position) being the most active as shown in the results.

VPA for fNIRS signals is widely used for the classification of active mental tasks (Hong and Khan, 2017; Khan and Hong, 2017; Zafar and Hong, 2018; Khan M.N.A. et al., 2020); however, less focus is observed on the use of VPA for classifying passive brain activities (Yoshino et al., 2013). This study focused on using the VPA for drowsiness classification while reducing the chances of false detection. Various statistical features like mean, slope, kurtosis, skewness, signal peak, and the sum of peaks of Δ*HbO*/Δ*HbR* signals (Khan and Hong, 2015; Naseer et al., 2016a; Hong et al., 2018; Khan R.A. et al., 2018; Nazeer et al., 2020a), as well as automatic feature extraction using deep learning networks (Khan R.A. et al., 2018; Tanveer et al., 2019; Wang et al., 2020), are previously used for classification of active as well as passive brain activities. VPA-based features like cerebral blood volume (Δ*CBV*), Δ*COE* and others are also used for active BCIs (Naseer et al., 2016b; Nazeer et al.,2020a,b). The best-performing statistical features (mean, peak, and the sum of peaks of Δ*HbO*) (Khan and Hong, 2015; Noori et al., 2017; Qureshi et al., 2017) along with VPA features are extensively tested in this study. Special focus is given to VPA-based features to prove the effective utilization of VPA for passive tasks. The classification results have shown confidence in using slopes of VPA indices as features. To avoid false detection, trends of trajectories are captured over a sample space of 5 s instead of using instantaneous values of hemodynamic signals as features. So the slopes of Δ*HbO*,Δ*HbR*,Δ*HbT*,Δ*COE*,|*R*|,∠*R* trajectories are true representative of changing brain state from wakefulness to drowsiness. The change in the trend of these factors checked against threshold criteria supports the earlier and actual estimation of drowsiness activity.

Threshold circles obtained by EEG response, the onset of tasks, resting state, baseline data, etc., are plotted upon VPA for the hemodynamic response, initial dip, activity detection, etc., in various fNIRS- and EEG-fNIRS-based hybrid BCI studies (Zafar and Hong, 2017; Hong et al., 2018; Khan M.J. et al., 2018; Nazeer et al., 2020a). This study employed threshold circles based upon sleep stages, which are not used before for fNIRS studies, up to the best knowledge of the authors. The novelty lies in proposing a uniform criterion that is readily applicable to any subject without requiring recalibration or extensive setup time. This feature increases the utility of this BCI scheme for practical applications and products.

General trends of the hemodynamic response of the brain during the transition from wakefulness to sleep have been investigated in fNIRS- and EEG-based BCI studies (Oniz et al., 2019). It intuits the use of hemodynamic response for sleep stage classification. The proposed threshold criteria are validated over the 13 subjects’ data with various feature sets and classifiers for multiclass classification. Results and performance measures of this tetra-class classification problem support the claim of this study for the wide applicability of proposed threshold criteria. Classification accuracy is more than 88.7% for all subjects, which is well above the chance rate (25%) for four-class classification and also more than 60% confidence level required for BCI utility (Asgher et al., 2020; Huang et al., 2020; Lindig-Leon et al., 2020). Adaptation of the system to any new subject requires only baseline data of wakefulness state, and other thresholds will be measured as proposed. This BCI scheme is promising for online detection systems.

## Conclusion

This study investigates the feasibility of the fNIRS-based passive BCI scheme for the detection of driver’s drowsiness and sleep stage classification. VPA along with fixed threshold circles is used for the online classification of this passive activity. Threshold circle criteria are based upon the CORE state of wakefulness and NREM sleep stages of any subject. The CORE trajectory, which is based upon both Δ*HbO* and Δ*HbR* indicators, is plotted upon VPA in real-time. The decision of drowsiness detection has occurred when the CORE trajectory crosses the threshold circles in the fourth quadrant of the vector phase diagram. To further validate the wide applicability of threshold circle criteria, extensive testing is done using various feature sets and classifiers over a dataset of 13 subjects. Results indicate that slopes of CORE vector angle and magnitude trajectories “*m*(∠*R*) and *m*(|*R*|)” are the best-suited features for drowsiness detection. The SVM classifier performed well overall with a mean classification accuracy of 92.5%, while the ensemble classifier took a minimum computation time of 44 ms for this four-class classification problem. Classification performance measures indicate that sleep stage-based threshold circle criteria are universally applicable for any subject with minimum setup time. Channel selection shows that the right DPFC is the more active region of the brain for drowsiness detection during driving tasks. This study validates a potential BCI scheme for real-time detection of passive brain responses for practical applications.

## Data Availability Statement

The datasets analyzed in this article are not publicly available. Requests to access the datasets should be directed to K-SH, kshong@pusan.ac.kr.

## Ethics Statement

The studies involving human participants were reviewed and approved by the Pusan National University Institutional Review Board. The patients/participants provided their written informed consent to participate in this study.

## Author Contributions

SA conceived the idea, processed the data, and wrote the first draft of the manuscript. MJK obtained the raw fNIRS data when he was a Ph.D. student at Pusan National University. NN developed the VPA method with a single-threshold circle. K-SH supervised the initial development of the VPA. HS and YA were involved in checking the results and manuscript. All authors have read and agreed to the published version of the manuscript.

## Conflict of Interest

The authors declare that the research was conducted in the absence of any commercial or financial relationships that could be construed as a potential conflict of interest.
